# Predicting Zoonotic Risk of Influenza A Viruses from Host Tropism Protein Signature Using Random Forest

**DOI:** 10.3390/ijms18061135

**Published:** 2017-05-25

**Authors:** Christine L. P. Eng, Joo Chuan Tong, Tin Wee Tan

**Affiliations:** 1 Department of Biochemistry, Yong Loo Lin School of Medicine, National University of Singapore, 117597 Singapore, Singapore; 2 Institute of High Performance Computing, A*Star, 138632 Singapore, Singapore; tongjc@ihpc.a-star.edu.sg; 3 National Supercomputing Centre, 138632 Singapore, Singapore; tinwee@nscc.sg

**Keywords:** influenza, zoonosis, machine learning

## Abstract

Influenza A viruses remain a significant health problem, especially when a novel subtype emerges from the avian population to cause severe outbreaks in humans. Zoonotic viruses arise from the animal population as a result of mutations and reassortments, giving rise to novel strains with the capability to evade the host species barrier and cause human infections. Despite progress in understanding interspecies transmission of influenza viruses, we are no closer to predicting zoonotic strains that can lead to an outbreak. We have previously discovered distinct host tropism protein signatures of avian, human and zoonotic influenza strains obtained from host tropism predictions on individual protein sequences. Here, we apply machine learning approaches on the signatures to build a computational model capable of predicting zoonotic strains. The zoonotic strain prediction model can classify avian, human or zoonotic strains with high accuracy, as well as providing an estimated zoonotic risk. This would therefore allow us to quickly determine if an influenza virus strain has the potential to be zoonotic using only protein sequences. The swift identification of potential zoonotic strains in the animal population using the zoonotic strain prediction model could provide us with an early indication of an imminent influenza outbreak.

## 1. Introduction

Influenza A viruses primarily reside in avian species, yet in recent years, there have been an increasing number of documented zoonotic infections in humans. After the first highly pathogenic H5N1 outbreak in 1997 in Hong Kong, there were subsequently many more local epidemic outbreaks from H5N1 viruses, especially in Asia and Africa [1,2,3]. There have also been a smaller number of human infections involving other avian influenza subtypes including H7N7 in United Kingdom and Netherlands [4,5,6], H9N2 in China [7,8], as well as the recent H7N9 outbreak in China [9,10]. Most of these zoonotic infections emerged in a similar manner, with patients having contracted the virus upon direct contact with poultry or other avian species [2,11,12]. While there was no direct evidence of human transmissibility or stable adaptation in humans, many of these zoonotic infections particularly, of H5N1 and H7N9 subtypes, cause severe illnesses, with the mortality rate for H5N1 estimated to be as high as 60% [13]. These zoonotic strains originated from avian species, having acquired sufficient mutations or new segments from reassortment to overcome host range restriction and successfully cause infections in humans.

Despite many years of intensive research, current surveillance technologies for influenza viruses remain limited as there are still no reliable measures in predicting zoonotic strains that can cause the next zoonotic outbreak or pandemic. Current surveillance efforts focus on detection, assessment and response following an outbreak [14,15]. Antigenic and genetic characterization of the new strains by phylogenetic analyses with existing strains are performed to understand how the outbreak started as well as to formulate effective response and treatment [16,17]. There have been increasing efforts in surveillance recently, with disease surveillance in wild birds and poultry farms where influenza sequence data are collected and deposited online [18,19]. Yet, the computational methods to identify possible zoonotic strains remain rudimentary, with the reliance on host-associated genetic markers [20]. A number of avian- or human-specific residues at certain amino acid positions have been identified to differentiate between avian and human strains [21,22], most notably the polymerase basic protein 2 (PB2) E627K host range determinant which shows a strong selection for the amino acid lysine (K) in human strains and some zoonotic strains as opposed to glutamate (E) carried by avian strains [23,24]. More recent bioinformatics approaches have identified diversity motifs or combinations of interacting amino acid residues to distinguish between avian or human strains [25,26]. However, these approaches are context-specific and generally do not apply to novel influenza subtypes [20,27], because mutations identified as critical in a particular zoonotic event may or may not be detected in other events. The World Health Organization (WHO) and the United States Center for Disease, Control and Prevention (CDC) have in recent years introduced influenza risk assessment tools to evaluate potential pandemic risks of influenza A viruses circulating in animal species [28,29]. Both tools consist of several evaluation criteria in three categories of viral properties, population attributes, and ecology and epidemiology to characterize the risk of a virus. While the tools are comprehensive, several evaluation criteria such as antiviral treatment resistance, receptor binding properties, and lab animal transmission require time and extensive testing in the laboratory. As such, it is still a challenge to predict potential zoonotic strains based on sequence information alone. 

There are also attempts in the development of machine learning approaches to predict zoonotic transmission. Qiang and Kou first developed a computational prediction model based on an artificial neural network (ANN) to predict interspecies transmission of influenza A viruses based on molecular patterns found in protein sequences [30]. The model utilized a wavelet packet decomposition method to extract energy feature vectors from protein sequences in the training process, distinguishing avian species with the capability to cross host species barrier from those that do not possess the zoonotic capability. Another paper by Wang et al. also described a prediction model developed from a support vector machine (SVM) to classify avian and human influenza A sequences [31]. The model employed position-specific entropy profiles of avian and human protein sequences [21], which were then transformed into feature vectors encoded with amino acid physicochemical properties. Both prediction models apply protein sequences from six influenza inner proteins: three viral polymerases polymerase acidic protein (PA), polymerase basic protein 1 (PB1), and PB2, nucleoprotein (NP), non-structural protein 1 (NS1), as well as matrix protein 1 (M1). While both models reported high accuracy in prediction, the accuracies in predicting past zoonotic strains from influenza outbreaks have not been verified.

To achieve this goal, we have constructed a zoonotic strain prediction model using the machine learning classifier random forest, capable of predicting avian, human or zoonotic influenza virus strains in this study. Our previous work on host tropism of individual influenza virus proteins has resulted in the construction of a host tropism prediction system [32]. The system consists of individual protein prediction models of 11 influenza A virus proteins: hemagglutinin (HA), M1, matrix protein 2 (M2), neuraminidase (NA), NP, NS1, non-structural protein 2 (NS2), PA, PB1, accessory protein F2 translated from PB1 segment (PB1-F2) and PB2, which independently predicts avian or human host tropism of each protein based on protein sequences translated into amino acid physicochemical properties feature vectors. We next combined the protein prediction results into a host tropism protein signature for each influenza virus strain, which is defined as an influenza viral proteome profile of 11 independent host tropism predictions of avian or human influenza virus proteins. The host tropism protein signature analysis of 12,624 strains has led to the discovery of distinct host tropism protein signatures between avian, human and zoonotic strains [33]. Furthering this finding, we utilized the host tropism protein signatures to build a computational prediction model which is able to predict zoonotic strains capable of causing human infections. Instead of the conventional avian versus human strains approach generally adopted [21,22,25,26], we have defined zoonotic strains as a separate category distinct from typical avian and human strains, resulting in a three-class classification of avian, human and zoonotic strains. We then additionally validated the capability of the zoonotic strain prediction model whereby avian strains shown to be possible sources of zoonotic outbreaks by previous studies were accurately identified by the prediction model. This represents a significant validation to the capability of the zoonotic strain prediction model in using protein sequences to detect zoonotic strains that can lead to an influenza outbreak.

## 2. Results

### 2.1. Sufficient Distinction in Host Tropism Protein Signatures to Characterize Zoonotic Strains

**Figure 1:**
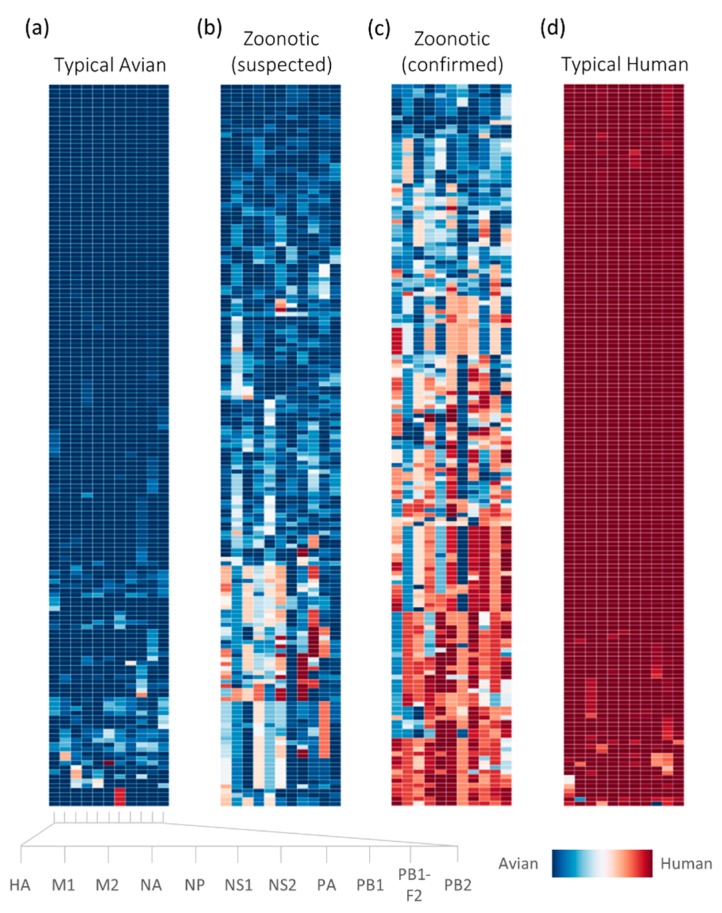
The dataset used in the study represented by host tropism protein signatures of influenza virus strains: 159 typical avian (**a**); 160 human-isolated confirmed zoonotic (**c**), and 164 typical human (**d**) strains were used in the construction of the zoonotic strain prediction model. Additionally, the signatures of a random 165 of 1047 avian-isolated suspected zoonotic strains (**b**) subsequently analyzed using the prediction model are also shown. Each individual virus strain in the bar is represented by the host tropism protein signature laid across the row, with the independent predictions of 11 proteins depicted in each column (HA, M1, M2, NA, NP, NS1, NS2, PA, PB1, PB1-F2, and PB2). Avian protein predictions are illustrated in blue, while human proteins are in red. The confidence of the avian or human host tropism prediction is expressed by the intensity of the color, based on the prediction probability estimates found in Supplementary S1 and S2 datasets. HA: hemagglutinin; M1: matrix protein 1; M2: matrix protein 2; NA: neuraminidase; NP: nucleoprotein; NS1: non-structural protein 1; NS2: non-structural protein 2; PA: polymerase acidic protein; PB1: polymerase basic protein 1; PB1-F2: accessory protein F2 translated from PB1 segment; PB2: polymerase basic protein 2.

Host tropism protein signatures obtained for the influenza virus strains in the dataset demonstrate the distinct signatures between avian, human and zoonotic strains. This is consistent with earlier findings where typical avian and human strains show almost unanimous host tropism predictions of avian or human proteins respectively, while suspected and confirmed zoonotic strains typically display a mixture of avian and human protein predictions [33]. As compared to the previous study however, the signatures generated in this study are of a higher resolution, owing to the avian and human probability distribution being used instead of binary predictions of either avian or human. Each host tropism protein prediction is associated with a probability estimate which represents the confidence of the prediction by each individual protein prediction model, loosely describing how “avian-like” or “human-like” the proteins are, as illustrated by the intensity of the color (Figure 1). This allows us to inspect with greater detail the host tropism protein signature of an influenza virus strain, which could provide a clue as to how much it has deviated from a typical strain.

The host tropism protein signatures indeed provided sufficient distinction for the classification of avian, human and zoonotic influenza virus strains. Based on the training samples in the dataset, the random forest zoonotic strain prediction model achieved very high prediction performance, 99.20% prediction accuracy and 1.000 weighted area under the receiver operating characteristic curve (AUC; Table 1). This represents the correct avian, human or zoonotic strain classification by the prediction model for 374 out of 377 strains in the training dataset. As identification of zoonotic strains are of greater emphasis in this study, the prediction accuracy for zoonotic strains, while slightly lower at a 98.40%, is still of satisfactory performance (Table 1). This could be attributed to the zoonotic strains having a much more diverse range of avian and human protein predictions in their signatures as compared to typical avian and human strains, hence amounting to the increase in difficulty to predict these strains. Nevertheless, the prediction performance by the random forest zoonotic strain prediction model is still significantly better than random three-class classification, highlighting that the host tropism protein signatures of zoonotic strains are sufficiently distinct from typical avian and human strains. This therefore enables the prediction model to identify zoonotic strains with a high standard of accuracy.

**Figure 2:**
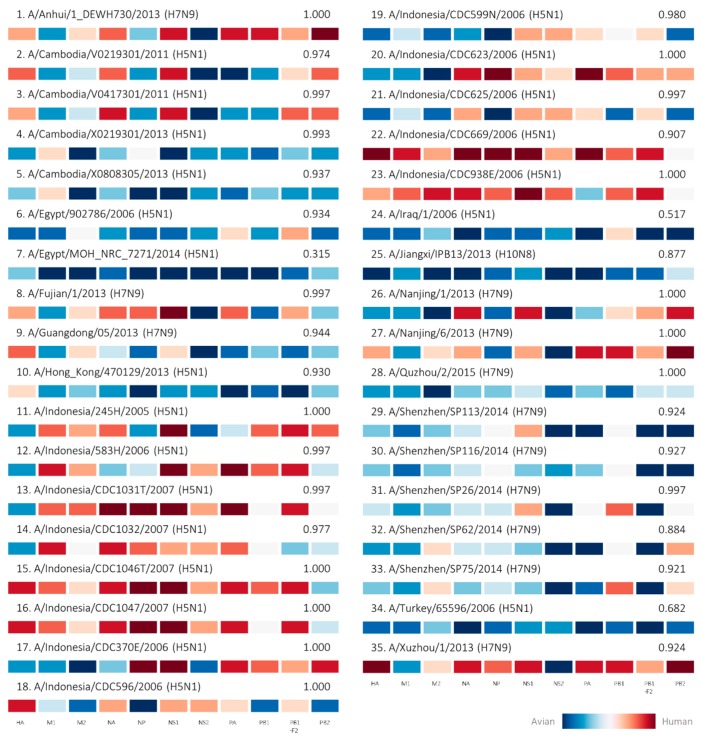
Human-isolated confirmed zoonotic strains prediction results by the prediction model during the independent validation process. A total of 35 zoonotic strains represented by their host tropism protein signatures were predicted by the zoonotic strain prediction model with the estimated zoonotic probability describing the confidence of the prediction. The strains are labelled with consecutive numbers for reference in the main text.

Independent validation of the prediction model with a separate testing dataset further affirms the high predictive performance of the model. The prediction model achieved a 99.06% prediction accuracy with 1.000 weighted AUC even when tasked to predict strains which were not included in the training process (Table 1). All but one of the zoonotic strains in the testing dataset including those isolated from H5N1 outbreaks in Asia and H7N9 outbreaks in China were correctly identified by the prediction model (Figure 2), resulting in a zoonotic prediction accuracy of 97.14% (Table 1). Results from this demonstrate that the prediction model was able to predict novel avian, human and zoonotic strains with high accuracy from the host tropism protein signature, even when presented with a diverse range of signatures. Most of the zoonotic strains were predicted with high zoonotic probabilities exceeding 0.8, with the remaining predicted with low to moderate zoonotic probabilities of 0.517 to 0.682, as well as one incorrect avian prediction with 0.315 zoonotic probability. What came as a surprise were the zoonotic strains which carried signatures of all avian tropism yet predicted accurately as zoonotic strains by the prediction model. This seems to suggest that zoonotic strains need not acquire human proteins to cause human infections. Results from this independent validation of the prediction model thus substantiate the capability of the model to accurately identify zoonotic strains in the future.

### 2.2. Retrospective Analysis of Avian Strains from Outbreaks Demonstrate Capability of Zoonotic Strain Prediction Model

**Figure 3:**
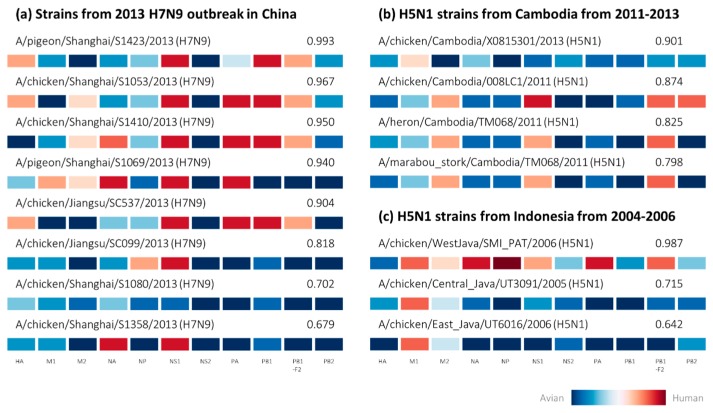
Avian-isolated strains from zoonotic influenza outbreaks represented by host tropism protein signatures with estimated zoonotic probability predicted by the zoonotic strain prediction model. The strains were cross-referenced with studies on phylogenetic analyses, demonstrating that some of these strains share high sequence similarities with zoonotic strains isolated from human patients during influenza outbreaks of (**a**) novel outbreak of H7N9 in China in 2013 and (**b**) H5N1 outbreaks in Cambodia from 2011 to 2013 as well as (**c**) Indonesia from 2004 to 2006.

By employing the zoonotic strain prediction model to perform an analysis of avian strains isolated from zoonotic outbreaks, we validated the capability of the prediction model to identify potential zoonotic strains circulating in avian species. Early studies on phylogenetic analyses from the H7N9 outbreak in China identified several avian-isolated strains sharing almost identical sequences with strains isolated from one of the first few human infections at the start of the outbreak [34,35], two of which in our dataset were successfully predicted as zoonotic by the prediction model with estimated zoonotic probabilities of 0.967 and 0.940 (Figure 3a). This corroborates earlier findings that the H7N9 outbreak originated from poultry and avian sources [34,35,36], as many strains isolated from avian species subsequently during the outbreak display classic zoonotic host tropism protein signatures with very high estimated zoonotic probabilities predicted by the prediction model. This would suggest that zoonotic H7N9 viruses had circulated among many avian species, covertly asymptomatic [35,36], to spread across China causing severe human infections in many states.

We also cross-referenced an additional six avian-isolated H7N9 strains from a recent study investigating the import of H7N9 human infections into Taiwan [37], which were all predicted as zoonotic by the prediction model. Four of the six strains were predicted with high zoonotic probabilities exceeding 0.8, while the remaining two strains were predicted with moderate zoonotic probabilities of 0.679 to 0.702. Intriguingly, we again observe a strain having all avian proteins in its host tropism protein signature being predicted as zoonotic with 0.702 zoonotic probability (Figure 3a). While we cannot confirm if this strain did indeed cause human infections during the outbreak, it is possible that it may evolve to be more zoonotic based on previous observation of several confirmed zoonotic strains also carrying all avian signatures (Figure 1).

**Figure A1:**
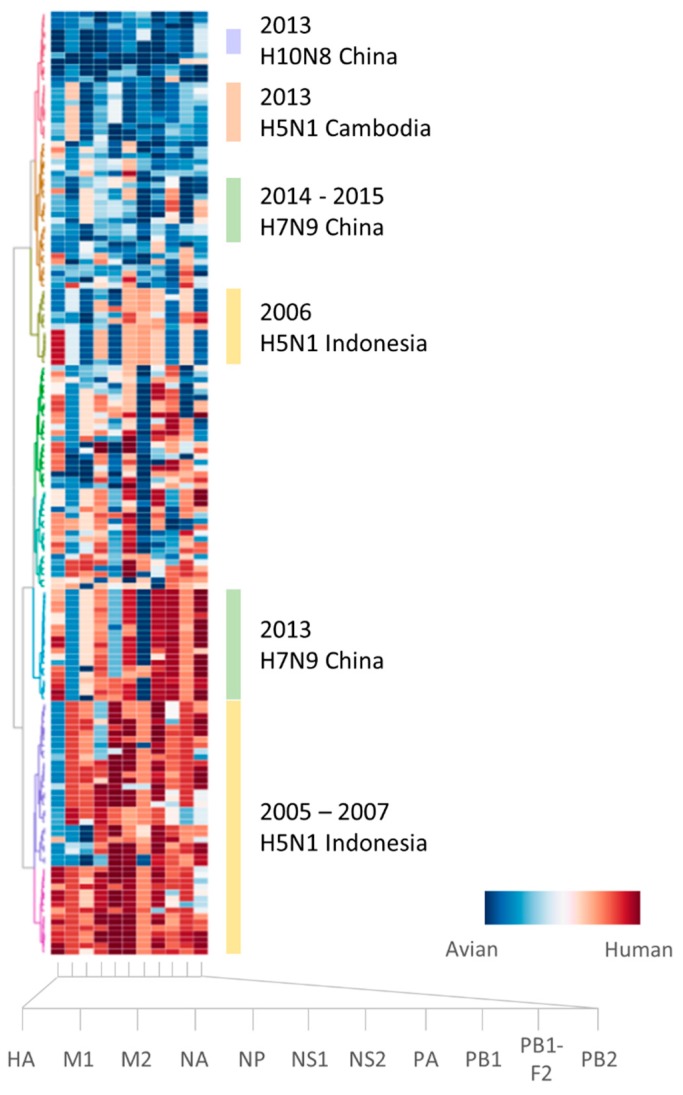
Hierarchical clustering of host tropism protein signatures of 160 confirmed zoonotic strains. Zoonotic strains from the same influenza outbreaks typically share similar signatures. Each row depicts the signature of a zoonotic strain, with host tropism predictions of 11 proteins shown in each column (HA, M1, M2, NA, NP, NS1, NS2, PA, PB1, PB1-F2, and PB2). Avian protein predictions are illustrated in blue while human proteins are in red. The confidence of the avian or human host tropism prediction is expressed by the intensity of the color, based on the prediction probability estimates found in Supplementary S1 and S2 datasets.

An additional four avian-isolated strains from Cambodia predicted as zoonotic by the prediction model (Figure 3b) were also observed from phylogenetic analyses of another study to share the same clades as human-isolated strains from H5N1 outbreaks in Cambodia from 2011 to 2013 [38]. Surprisingly, closer observation shows that the host tropism protein signature of the strain with the highest zoonotic risk of 0.901 actually contains the least number of human proteins among all four Cambodian strains (Figure 3b), with only the M1 protein having slight human tropism. Indeed, a cross examination showed that the zoonotic strains isolated from human patients during the H5N1 outbreak in Cambodia carried similar host tropism protein signatures (Appendix A Figure A1). This demonstrates that zoonotic strains from the same outbreaks carried similar host tropism protein signatures.

Results from our analysis also suggest that not all influenza viruses isolated from avian species during the outbreaks are zoonotic strains capable of causing human infections. Of the three H5N1 strains isolated from chicken in Indonesia, one was predicted with very high zoonotic probability of 0.987, with the remaining two strains predicted with lower zoonotic probability estimates of 0.642 and 0.715 (Figure 3c). Phylogenetic analysis of the strains by another study demonstrated the close evolutionary relationships of the HA and NA glycoproteins to confirmed zoonotic strain isolated from human patients in 2005 [39,40]. Nevertheless, this indicates that not all avian species or poultry sources were infected with zoonotic strains, as some avian strains of the same subtype circulating in the same region might in fact not have the capability to cause human infections. Taken together, this might present an exciting prospect in the future where we can monitor avian influenza strains to determine their zoonotic risks in causing human infections.

## 3. Discussion

This study describes the successful use of machine learning on influenza sequence data to predict avian-to-human transmission of influenza viruses. Using host tropism protein signatures of influenza viruses which are predicted from protein sequences, the zoonotic strain prediction model can accurately distinguish between typical avian strains found in avian species, seasonal influenza circulating in humans, and zoonotic strains originating from avian species that have caused human infections. Almost all known zoonotic strains from past influenza outbreaks with complete proteome were accurately predicted as zoonotic, regardless of their HA and NA subtypes, which also includes the less common subtype of H10N8 in addition to H5N1 and H7N9 subtypes (Figure 2). As compared to the context-specific application of host-associated genetic markers, the zoonotic strain prediction model is not restricted by this limitation and can be applied for prediction across all influenza subtypes.

The design of this study employs a systems approach which includes two layers of machine learning on influenza protein sequences to predict zoonotic strains. This is a departure from most other studies looking into avian- and human- specific amino acid residues which are primarily based on sequence alignments of common influenza subtypes such as H1N1, H3N2 and H5N1 [21,22], as well as machine learning approaches to predict avian or human sequences using the host-specific residues [25,31]. Here, we defined zoonotic strains as a third, separate category in addition to avian and human strains. These zoonotic strains are recognized as an intermediate between avian and human strains, where they may have started evolving to overcome the host species barrier, but have not adequately adapted to humans yet. These changes can be reflected in the mixture of avian and human proteins in their host tropism protein signatures (Figure 1). By using the host tropism protein signatures which are in turn host tropism predictions based on global amino acid physicochemical properties descriptors, the zoonotic prediction model can recognize potential zoonotic strains, regardless of subtypes. This is in contrast with conventional approaches investigating amino acid positions which show strong selection for either avian or human strains. While the host-associd genomic markers are useful in providing clues behind the mechanism of avian-to-human transmission, the zoonotic prediction model constructed in this study aims to complement existing tools by providing a rapid prediction of zoonotic strains using a machine learning approach. This would allow a swift detection of possible zoonotic strains circulating in avian species, which can then be further analyzed for their host-associated genomic markers.

Analysis of the zoonotic strains using the zoonotic strain prediction model illustrates that zoonotic events are truly a complex process. Similar to results from our previous study [33], there are no universal host tropism protein signatures for zoonotic strains with strains from the same influenza outbreaks sharing similar signatures (Appendix A Figure A1). Additionally, we have encountered some puzzling observations from the analysis of the host tropism protein signatures, where some zoonotic strains were observed to be carrying all avian tropism in their signatures. As these confirmed zoonotic strains isolated from human patients carrying predominantly avian signatures can also be found in the training dataset (Appendix A Figure A1), this resulted in high zoonotic probability predictions by the zoonotic strain prediction model in the testing phase (Figure 2) as well as the subsequent analysis for similar strains (Figure 3). This is due to the supervised training process by the machine learning classifier where the prediction model learns from training examples provided in the training dataset. Nevertheless, it can be observed crudely that the avian proteins for these zoonotic strains seem to carry less avian tropism compared to typical avian strains (Figure 1). Despite that, our goal in this study is to use protein sequences to detect zoonotic strains that pose a risk to cause human infections circulating in avian species, and we have successfully shown with these findings the potential of the zoonotic strain prediction model in using the underlying host tropism protein signatures to predict zoonotic strains with a high degree of accuracy. We aim to provide a tool capable of predicting zoonotic strains circulating in avian species rapidly using only protein sequences. Understanding how zoonotic strains are generated to cause outbreaks however, is a subject of further intensive investigation, requiring much more data on zoonotic strains with the aid of in-depth phylogenetic analysis.

While we are no closer to dissecting exactly which proteins are required for zoonotic influenza strains to make that zoonotic leap, we are slowly beginning to understand that we need to approach the problem from a systems perspective by looking at contributions of all influenza virus proteins. In using the host tropism protein signature, it is now possible to predict zoonotic strains as well as estimate the zoonotic risk. Nonetheless, the zoonotic prediction model is still in its infancy stages with limited data on confirmed zoonotic strains owing to the scarcity of complete genomic sequences from earlier zoonotic outbreaks. This is evident from the training dataset containing only zoonotic strains isolated since the beginning of the 21st century (Supplementary S1 dataset), as zoonotic strains prior to that do not have the complete the host tropism protein signature required for prediction. The strength of the zoonotic strain predictor lies in taking into consideration the contribution of all influenza virus proteins and by using them to distinguish between avian, human and zoonotic strains.

The zoonotic strain prediction model has been validated with confirmed zoonotic strains from past influenza outbreaks, where most zoonotic strains were predicted correctly as zoonotic with high zoonotic probability estimates. Due to the probabilistic nature of the random forest classifier with the classification output determined by majority voting of the random trees in the forest [41], it is possible to manually define a threshold for the probability estimates in each classification. Based on the predicted zoonotic probability estimates from the confirmed zoonotic strains in the independent testing dataset as well as the analysis from the avian-isolated suspected zoonotic strains, we propose a zoonotic risk table to aid in the interpretation of each strain prediction by the zoonotic strain prediction model (Table 2). This is again in preliminary stages based on the data in this study, with the sensitivity of zoonotic strain detection at 0.988 by defining the threshold for zoonotic probability estimate at 0.7. With the increase in influenza surveillance and sequencing of complete genome in the future, the zoonotic strain prediction model can only improve with the collection of more data for continuous training of the prediction model. This would also help us further understand which proteins are required for interspecies transmission. In the meantime, the zoonotic strain prediction model could prove to be a valuable addition to influenza virologic surveillance to complement traditional analytical methods through the monitoring of influenza strains in avian species and poultry, and by providing swift prediction on the zoonotic risks of influenza virus strains using sequence data.

## 4. Materials and Methods

### 4.1. Data Collection and Preparation

Influenza A virus protein sequence data was acquired from Influenza Research Database (http://www.fludb.org (accessed on 27 October 2015)) [42]. The data was next processed to retain only influenza A virus strains with complete proteome, comprising complete full-length sequences of 11 proteins (HA, M1, M2, NA, NP, NS1, NS2, PA, PB1, PB1-F2, PB2). This included the removal of invalid protein sequences with non-standard amino acids or of incomplete lengths, as well as the removal of strains with multiple contradictory sequences of the same protein. The complete dataset consisted of 13,998 strains with 7592 avian strains and 6406 human strains.

We next identified zoonotic strains which were distinct from typical avian and human strains. Based on published literature on avian or zoonotic influenza outbreaks, WHO reports and CDC reports, a total of 160 confirmed zoonotic strains were identified from strains isolated from human cases during influenza outbreaks from 1997 to 2015 (Appendix A Table A1) [3,4,5,6,7,8,9,10,38,43,44,45,46,47,48,49,50,51,52,53,54,55,56,57,58,59,60,61,62,63,64,65,66,67,68]. An additional 1047 avian-isolated strains collected during the same period as the outbreaks around the geographic region were also identified and designated as avian-isolated suspected zoonotic strains. The dataset used in this study is thus categorized into three groups of avian, human and zoonotic strains, with the avian-isolated suspected zoonotic strains excluded from the following prediction model construction process for subsequent analysis.

### 4.2. Host Tropism Protein Signature Feature Transformation

Host tropism protein signatures for all influenza strains were next obtained using the host tropism protein prediction system (http://fluleap.bic.nus.edu.sg (accessed on 7 December 2015)) [32]. The system provides independent avian or human host tropism predictions of 11 influenza virus proteins. For each individual protein, the host tropism prediction model predicts avian or human host tropism based on protein sequence input. The protein sequences were represented by 146 feature vectors comprising 20 standard amino acid compositions and global descriptors of six amino acid physicochemical properties of hydrophobicity, normalized van der Waals volume, polarity, polarizability, charge and solvent accessibility [32]. Based on these, the avian or human host tropism prediction results for 11 proteins are integrated as a host tropism protein signature for each strain. Each prediction by the respective protein prediction model in the system is predicted with avian and human probability distribution to indicate the confidence of host tropism prediction based on the protein sequence. In summary, each influenza virus strain is thus represented by 22 avian and human probability distributions of 11 host tropism predictions of each protein (Figure 1). The 22 avian and human probability distributions therefore compose the training dataset for the subsequent machine learning process.

### 4.3. Construction of Zoonotic Strain Prediction Model

The influenza virus strains represented by the host tropism protein signatures were next used for machine learning to build a zoonotic strain prediction model in the classification of three groups of avian, human and zoonotic strains. The zoonotic strains in this training process consist only of the confirmed zoonotic strains isolated from human patients during influenza outbreaks. As the number of avian and human strains were disproportionately greater than the number of confirmed zoonotic strains, the method of down-sampling was introduced to prevent an imbalanced dataset. An imbalanced dataset may result in bias in the training process which may affect the performance evaluation. In the down-sampling process, avian and human strains were randomly removed to result in approximate equal number of strains in the three groups of avian, human and zoonotic strains. Following that, the final dataset was partitioned into separate training (80%) and testing (20%) datasets (Table 3).

The machine learning algorithm employed in the construction of the prediction model is random forest. Random forest is an ensemble of decision trees, where the random trees are grown using the bagging technique, in which a randomly selected subset of features from the entire feature space is selected to split each leaf node in the tree [41]. Random forest has been shown to consistently achieve high performance and is also the most suited for this as the dataset was obtained from host tropism predictions made on random forest protein prediction models as well [32]. This was performed on the WEKA machine learning platform [69], the Waikato Environment for Knowledge Analysis software containing a suite of machine learning algorithms for data mining and classification tasks. Ten-fold cross-validation training was applied to minimize the effect of overfitting. In this process, the training dataset is randomly partitioned into nine training subsets and one testing subset over ten iterations. The algorithm will train with nine training subsets and evaluate the prediction model with the remaining testing subset for every iteration, with each subset used exactly once as testing. Results for the performance evaluation are taken as an average of ten iterations, and the model with the best results is chosen.

In addition, a parameter optimization process was also performed in the training process. The optimized random forest parameters were the number of trees in the random forest and the number of features to use in random selection. These were fine-tuned using the grid search approach where each parameter in a manually defined subset of a maximum of 500 trees and 22 features, is exhaustively applied to select for the parameters producing the best results. This approach ensures that the best parameters were chosen to maximize the performance in constructing the prediction model. The final random forest prediction model was constructed with 302 trees in the random forest, with 1 random feature at each branch split.

The prediction model was next assessed with several performance measures. This includes overall prediction accuracy and AUC. The prediction accuracy measures the number of predictions correctly made from the total number of strains in the training dataset. AUC, on the other hand, describes the probability of a randomly chosen positive sample ranking higher than a randomly chosen negative sample by the model [70,71]. As this study involves a three-class classification problem with an approximately balanced dataset, the models were evaluated primarily with overall prediction accuracy and weighted AUC of the three groups of avian, human and zoonotic prediction. Furthermore, the prediction accuracy and AUC for zoonotic strains were also taken into account as the primary concern of this study is in the prediction of zoonotic strains. This was implemented through the generalization of the three-class classification into a binary classification of zoonotic versus non-zoonotic comprising both avian and human strains.

The completed zoonotic strain prediction model was finally independently validated with the testing dataset, consisting of strains which were excluded from the initial training process. Performance of the model in predicting strains from the separate testing dataset could help establish whether overfitting has occurred in the training process. This would hence determine if the model is robust for accurate prediction of novel strains in the future.

The zoonotic strain prediction model classifies a strain as avian, human, or zoonotic from the feature vectors represented by the host tropism protein signatures. The random forest algorithm is, by nature, a probabilistic classifier where the outputs are continuous decision values determined based on voting by the random trees in the random forest [41]. Therefore, each strain prediction by the random forest prediction model has an avian, human and zoonotic probability estimate as calculated from the number of votes by the random trees out of the total number of trees in the forest. This represents the confidence of the prediction by the random forest prediction model. The final predicted avian, human or zoonotic classification of a strain would thus be the class with the highest probability estimate.

### 4.4. Analysis of Avian-Isolated Suspected Zoonotic Strains

The zoonotic strain prediction model was then tasked to analyze the zoonotic risks of avian-isolated suspected zoonotic strains. This group of strains were excluded initially from both the training and testing process as not all the avian strains isolated from influenza outbreaks contributed to the onset of the outbreak [40]. Thus, the zoonotic capability of these strains cannot be established with certainty. The strains, represented by their host tropism protein signatures, were provided to the zoonotic strain prediction model for prediction. The resulting avian, human or zoonotic classifications, along with the estimated probability distributions, were analyzed in conjunction with the host tropism protein signatures.

## 5. Conclusions

Our study demonstrated the successful use machine learning trained on host tropism protein signatures to predict zoonotic strains having the capability to cause human infections. The zoonotic strain prediction model is proposed as an influenza virologic surveillance tool to detect changes in protein sequences in avian strains that may indicate a zoonotic jump event. As influenza sequence data are already regularly sampled and collected [18,19], this tool could complement existing methods to rapidly screen for possible zoonotic strains. Future work to integrate geographical and ecological data [72] would bring more significant advancements in predicting future influenza outbreaks beyond current sequence prediction capabilities. The detection of possible zoonotic strains in avian species in the future could grant us precious time in formulating appropriate responses before they can reach the human population to start devastating outbreaks. This would ultimately not only benefit public health, but also reduce the economic impact to the agriculture industry in the event of an influenza outbreak. The zoonotic strain prediction model is available for prediction online at http://fluleap.bic.nus.edu.sg (accessed on 20 May 2017).

## Figures and Tables

**Table 1:** Performance evaluation of random forest zoonotic strain prediction model. AUC: area under the receiver operating characteristic curve.

Stage	Overall Accuracy	Weighted AUC	Zoonotic Accuracy	Zoonotic Sensitivity	Zoonotic Specificity	Zoonotic AUC
Training	99.20	1.000	98.40	0.984	0.996	1.000
Testing	99.06	1.000	97.14	0.971	1.000	0.999

**Table 2:** Proposed zoonotic risk interpretation table based on zoonotic probability estimates predicted by the zoonotic strain prediction model.

Zoonotic Probability Estimate	Zoonotic Risk
≥0.7	High
<0.7	Low to none

**Table 3:** Final dataset of avian, human and zoonotic strains for construction of the zoonotic strain prediction model.

Category	Training Dataset	Testing Dataset	Total Samples in Group
Avian	125	34	159
Human	127	37	164
Zoonotic	125	35	160
Total samples in dataset	377	106	483

## Supplementary Materials

Supplementary materials can be found at www.mdpi.com/1422-0067/18/6/1135/s1. Dataset S1. Training dataset for the zoonotic strain prediction model. Dataset S2. Independent testing dataset for the zoonotic strain prediction model. Dataset S3. Dataset for analysis of avian-isolated suspected zoonotic strains using the zoonotic strain prediction model.

## Abbreviations

ANN	Artificial neural network
AUC	Area under the receiver operating characteristic curve
CDC	Center for Control, Disease and Prevention
HA	Hemagglutinin
M1	Matrix protein 1
M2	Matrix protein 2
NA	Neuraminidase
NP	Nucleoprotein
NS1	Non-structural protein 1
NS2	Non-structural protein 2
PA	Polymerase acidic protein
PB1	Polymerase basic protein 1
PB1-F2	Accessory protein F2 translated from PB1 segment
PB2	Polymerase basic protein 2
SVM	Support vector machine
WHO	World Health Organization

## Appendix A

**Table A1:** Total number of suspected and confirmed zoonotic strains with complete proteome identified from zoonotic influenza outbreaks.

Year	Subtype	Country	Avian-isolated Suspected Zoonotic Samples	Human-Isolated Confirmed Zoonotic Samples
1997	H5N1	Hong Kong		
	H9N2	Hong Kong	2	
1998	H9N2	China	1	
1999	H9N2	China	2	
2003	H5N1	China	32	
		Vietnam		
	H7N7	Netherlands	1	
	H9N2	Hong Kong	2	
2004	H5N1	Thailand	10	
		Vietnam	14	
	H7N3	Canada	1	1
2005	H5N1	Cambodia		
		China	2	
		Indonesia	11	8
		Thailand	22	
		Vietnam	46	
2006	H5N1	Azerbaijan		
		Cambodia		
		China	19	
		Djibouti		
		Egypt	4	2
		Indonesia	11	46
		Iraq		1
		Thailand	7	1
		Turkey		3
2007	H3N8	Laos	1	
	H5N1	Cambodia		
		China	7	
		Egypt	2	
		Indonesia	4	10
		Laos	16	
		Myanmar		
		Nigeria	20	
		Pakistan		
		Vietnam	54	
	H7N2	United Kingdom		
2008	H3N8	Vietnam	1	
	H5N1	Bangladesh		1
		Cambodia		
		China	5	
		Egypt	18	
		Indonesia	1	
		Vietnam	4	
	H9N2	Hong Kong	9	
	H11N9	Vietnam	2	
2009	H5N1	Cambodia	1	
		China	6	
		Egypt	6	
		Indonesia	1	
		Vietnam		
	H9N2	Hong Kong	4	
2010	H5N1	Cambodia	5	1
		China	3	1
		Egypt	25	
		Indonesia		
		Vietnam	3	
2011	H5N1	Bangladesh	1	1
		Cambodia	6	4
		China	11	
		Egypt	13	
		Indonesia		
2012	H5N1	Bangladesh	18	
		Cambodia	1	3
		China	3	
		Egypt	6	
		Indonesia		
	H5N1	Vietnam	30	
	H7N3	Mexico		1
2013	H5N1	Bangladesh	5	
		Cambodia	8	10
		Canada		
		China	3	
		Egypt	3	
		Indonesia		
		Vietnam	19	
	H7N9	China	114	35
		Hong Kong		1
		Taiwan		2
	H9N2	China	135	1
	H10N8	China	26	4
2014	H5N1	Cambodia		
		China	7	
		Egypt	3	1
		Indonesia		
		Vietnam	24	
	H7N9	China	226	19
2015	H5N1	China		
		Egypt		
		Indonesia		
	H5N6	China		1
	H7N9	Canada		
		China		2
		Total	1047	160
